# Combined analysis of preoperative and postoperative lymphocyte-C-reactive protein ratio precisely predicts outcomes of patients with gastric cancer

**DOI:** 10.1186/s12885-022-09716-9

**Published:** 2022-06-11

**Authors:** Kozo Miyatani, Shohei Sawata, Masahiro Makinoya, Wataru Miyauchi, Shota Shimizu, Yuji Shishido, Tomoyuki Matsunaga, Manabu Yamamoto, Naruo Tokuyasu, Shuichi Takano, Teruhisa Sakamoto, Toshimichi Hasegawa, Hiroaki Saito, Yoshiyuki Fujiwara

**Affiliations:** 1 Department of Surgery, Division of Gastrointestinal and Pediatric Surgery, School of Medicine, Tottori University Faculty of Medicine, 36-1 Nishi-cho, Yonago, 683-8504 Japan; 2 Department of Surgery, Japanese Red Cross Tottori Hospital, Tottori, 680‑8517 Japan

**Keywords:** Gastric cancer, LCR, Prognosis

## Abstract

**Background:**

The systemic inflammatory response resulting from the complex interactions between cancer and the host plays an important role in cancer development. Recently, the lymphocyte-C-reactive protein ratio (LCR), which is a hematological and biochemical marker that reflects the systemic inflammatory response and nutritional status, has been reported to be associated with poor survival. Similar results were observed in patients with certain cancer types. However, these studies focused on the preoperative LCR, and thus far, no studies have reported the relationship between postoperative LCR and prognosis in patients with gastric cancer (GC).

**Methods:**

This study enrolled 455 patients with a histopathological diagnosis of gastric adenocarcinoma who underwent curative surgery at our institution between 2005 and 2018. The relationship between both the preoperative and postoperative LCR and the prognosis of patients with GC was retrospectively investigated.

**Results:**

Preoperative LCR showed significant correlations with tumor-related factors, such as tumor size, depth of invasion, and lymph node metastasis. By contrast, no correlation was observed between postoperative LCR and tumor-related factors. The 5 year survival rate was significantly worse in patients with low preoperative LCR than in those with high preoperative LCR (65.4% vs. 83.9%, *p* < 0.0001). Similarly, the 5 year survival rate was also significantly worse in patients with low postoperative LCR than in those with high postoperative LCR (67.0% vs. 84.1%, *p* < 0.0001). Furthermore, combination analysis of the pre- and postoperative LCR revealed that the prognosis of patients with both low pre- and postoperative LCR was worse in patients with GC (5 year survival rate was 52.0%). A multivariate analysis indicated that a low pre- and postoperative LCR and age and lymph node metastasis were independent prognostic indicators.

**Conclusions:**

The combination of preoperative and postoperative LCR appears to be useful in predicting the prognosis of patients with GC.

## Background

Worldwide, over 1,000,000 new gastric cancers (GC) cases and 783,000 GC deaths were estimated to have occurred in 2018, and thus, GC ranks as the fifth most frequently diagnosed cancer and the third leading cause of cancer mortality [1]. Therefore, determining the postoperative prognosis of patients with GC is crucial. When considering the prognosis of patients with malignant tumors, the TNM-classification system [2], which considers tumor-related factors and accurately reflects prognosis, has been widely used. Several studies of patients with GC have demonstrated that the depth of invasion and presence or absence of lymph node metastasis may be considered the most important prognostic factors [3, 4]. On the contrary, over the past few years, many researchers have suggested that the outcomes of patients with cancer are determined not only by tumor-related factors but also by patient-related factors, including inflammation, nutrition, and immune status. Recently, many studies have shown the prognostic significance of certain host-related factors based on systemic inflammation, such as the neutrophil–lymphocyte ratio (NLR), the platelet-lymphocyte ratio (PLR), and the Glasgow Prognostic Score (GPS), which are independent prognostic factors of survival in patients with GC [5–7]. Additionally, a chronic systemic inflammatory response is clearly associated with the progressive nutritional decline seen in patients with cancer and their subsequent poor outcomes [8]. For example, the prognostic nutritional index (PNI) was reported as a prognostic indicator in patients after radical resection for GC [9]. However, most of these reports explored the preoperative status, and few studies have been conducted to investigate the prognostic impact of the postoperative status. Moreover, we reported that markers of postoperative inflammation and nutrition, such as the postoperative NLR [10] and the postoperative PNI [11], are also related to the prognosis of patients with GC. Recently, Okugawa et al. reported the preoperative LCR as a novel nutrition-inflammation marker that predicts prognosis and the risk of postoperative surgical site infection in patients with GC [12]. Additionally, several studies have shown that the LCR can predict oncological outcomes in some types of malignancies [13–15]. However, no reports have been published on the relationship between postoperative LCR, which may represent the systemic inflammatory response and the nutritional status after tumor removal, and the prognosis in patients with GC. The aim of the present study was to determine the prognostic significance of preoperative and postoperative LCR in patients with GC.

## Methods

### Patients

This study enrolled 455 patients with a histopathological diagnosis of gastric adenocarcinoma who underwent curative surgery at Tottori University Hospital between 2005 and 2018. The data were collected retrospectively. Clinicopathological findings were generally determined according to the 15th edition of the Japanese Classification of Gastric Carcinoma [16]. All patients underwent either distal or proximal partial or total gastrectomy with regional lymph node dissection. We collected data from blood tests conducted preoperatively and 1 month postoperatively. The LCR was then calculated as follows: total lymphocyte count (LC) (number/μL)/ C-reactive protein (CRP) level (mg/dL). Postoperative complications were considered grade 2 or higher according to the Clavien–Dindo classification. Patients were periodically checked for early recurrence by diagnostic imaging (chest X-ray, double-contrast barium meal study, upper gastrointestinal endoscopy, ultrasonography, and computed tomography). Causes of death were determined by reviewing the medical records, which included laboratory data, ultrasonography, computed tomography, scintigrams, peritoneal punctures, and laparotomies, or by direct inquiry with family members. In some cases, postmortems were conducted to determine the cause of death. Institutional review board of Tottori University Hospital approved this study and waived informed consent.

### Statistical analysis

For statistical analyses, chi-square and Fisher’s exact probability tests were used to compare the distribution of individual variables between patient groups. Differences between the two groups were evaluated using the Mann–Whitney U test. Survival curves were calculated according to the Kaplan–Meier method. Differences between survival curves were examined using the log-rank test. We conducted a multivariate analysis of factors considered to predict overall survival (OS) and relapse-free survival (RFS) using a Cox proportional hazards model. A *p* < 0.05 was considered significant. All statistical analyses were conducted with EZR (Saitama Medical Center, Jichi Medical University, Saitama, Japan), which is a graphical user interface for R (The R Foundation for Statistical Computing, Vienna, Austria). More precisely, EZR is a modified version of R commander designed to add statistical functions frequently used in biostatistics.

## Results

The mean preoperative and postoperative LCR values were 39,650 (range: 190–347,900) and 41,487 (range: 132–333,900), respectively. Table 1 shows the correlations between the preoperative and postoperative LCR and clinicopathological variables in patients with GC. Statistically significant correlations were found between low preoperative LCR and age (*p* < 0.0001), gender (*p* = 0.001), tumor size (*p* = 0.002), depth of invasion (*p* = 0.006), lymph node metastasis (*p* = 0.013), lymphatic involvement (*p* = 0.006), vascular involvement (*p* = 0.025), lymphadenectomy (*p* = 0.003), and postoperative complications (*p* = 0.012). By contrast, statistically significant correlations were found between low postoperative LCR and age (*p* < 0.0001), gender (*p* = 0.0007), CEA level (*p* = 0.045), and postoperative complications (*p* < 0.0001).

**Table 1:** Relationships between the preoperative and postoperative LCR and clinicopathological variables in patients with gastric cancer

	Pre-LCR	*p* value	Post-LCR	*p* value
Age		< 0.0001		< 0.001
< 75 (*n* = 298)	45,508 ± 52,948		49,304 ± 55,861	
≥ 75 (*n* = 157)	28,532 ± 36,722		26,652 ± 35,818	
Gender		0.001		0.0007
Male (*n* = 332)	34,784 ± 44,367		36,508 ± 45,063	
Female (*n* = 123)	52,786 ± 56,686		54,928 ± 62,532	
Tumor size		0.002		0.294
< 4 cm (*n* = 277)	44,611 ± 53,185		43,890 ± 53,136	
≥ 4 cm (*n* = 178)	31,930 ± 39,383		37,749 ± 47,319	
Differentiation		0.421		0.289
Differentiated (*n* = 261)	37,479 ± 47,514		38,031 ± 46,889	
Poorly differentiated (*n* = 194)	42,571 ± 50,015		46,137 ± 55,790	
Depth of invasion		0.006		0.55
T1 (*n* = 287)	43,386 ± 48,998		43,027 ± 52,705	
T2/3/4 (*n* = 168)	33,268 ± 47,398		38,858 ± 47,912	
Lymph node metastasis		0.013		0.582
Absent (*n* = 325)	42,652 ± 49,065		42,533 ± 51,809	
Present (*n* = 130)	32,147 ± 46,790		38,874 ± 48,923	
Lymphatic involvement		0.006		0.183
Absent (*n* = 199)	46,685 ± 53,217		44,284 ± 51,689	
Present (*n* = 256)	34,182 ± 44,032		39,314 ± 50,405	
Vascular involvement		0.025		0.203
Absent (*n* = 229)	45,495 ± 53,053		42,729 ± 49,083	
Present (*n* = 226)	33,728 ± 42,956		40,230 ± 52,901	
Stage		0.063		0.681
I (*n* = 307)	41,935 ± 48,565		42,928 ± 52,670	
II/III (*n* = 148)	34,912 ± 48,517		38,500 ± 47,296	
CEA		0.184		0.045
< 5 ng/mL (*n* = 396)	41,296 ± 50,745		43,062 ± 51,413	
≥ 5 ng/mL (*n* = 59)	28,604 ± 28,539		30,920 ± 46,980	
CA19-9		0.285		0.571
< 37 ng/mL (*n* = 417)	40,670 ± 50,059		42,401 ± 52,312	
≥ 37 ng/mL (*n* = 38)	28,463 ± 25,994		31,464 ± 31,666	
Gastrectomy		0.516		0.796
Distal/proximal (*n* = 350)	40,438 ± 49,015		41,099 ± 49,566	
Total (*n* = 105)	37,026 ± 47,358		42,783 ± 55,645	
Lymphadenectomy		0.003		0.335
D0/D1/D1 + (*n* = 315)	43,373 ± 49,489		42,477 ± 50,033	
D2 (*n* = 140)	31,274 ± 45,632		39,262 ± 53,148	
Operation time		0.092		0.878
< Median (*n* = 227)	36,386 ± 44,043		42,071 ± 50,172	
≥ Median (*n* = 228)	42,900 ± 52,658		40,907 ± 51,865	
Postoperative complications		0.012		< 0.0001
Absent (*n* = 314)	43,607 ± 52,275		46,013 ± 51,861	
Present (*n* = 141)	30,839 ± 37,936		31,411 ± 47,591	

All results are expressed as the mean ± standard deviation

*CEA* Carcinoembryonic antigen, *CA19-9* Carbohydrate antigen 19–9

The median operation time was 324 min

Postoperative complications were considered grade 2 or higher according to the Clavien–Dindo classification

**Fig. 1:**
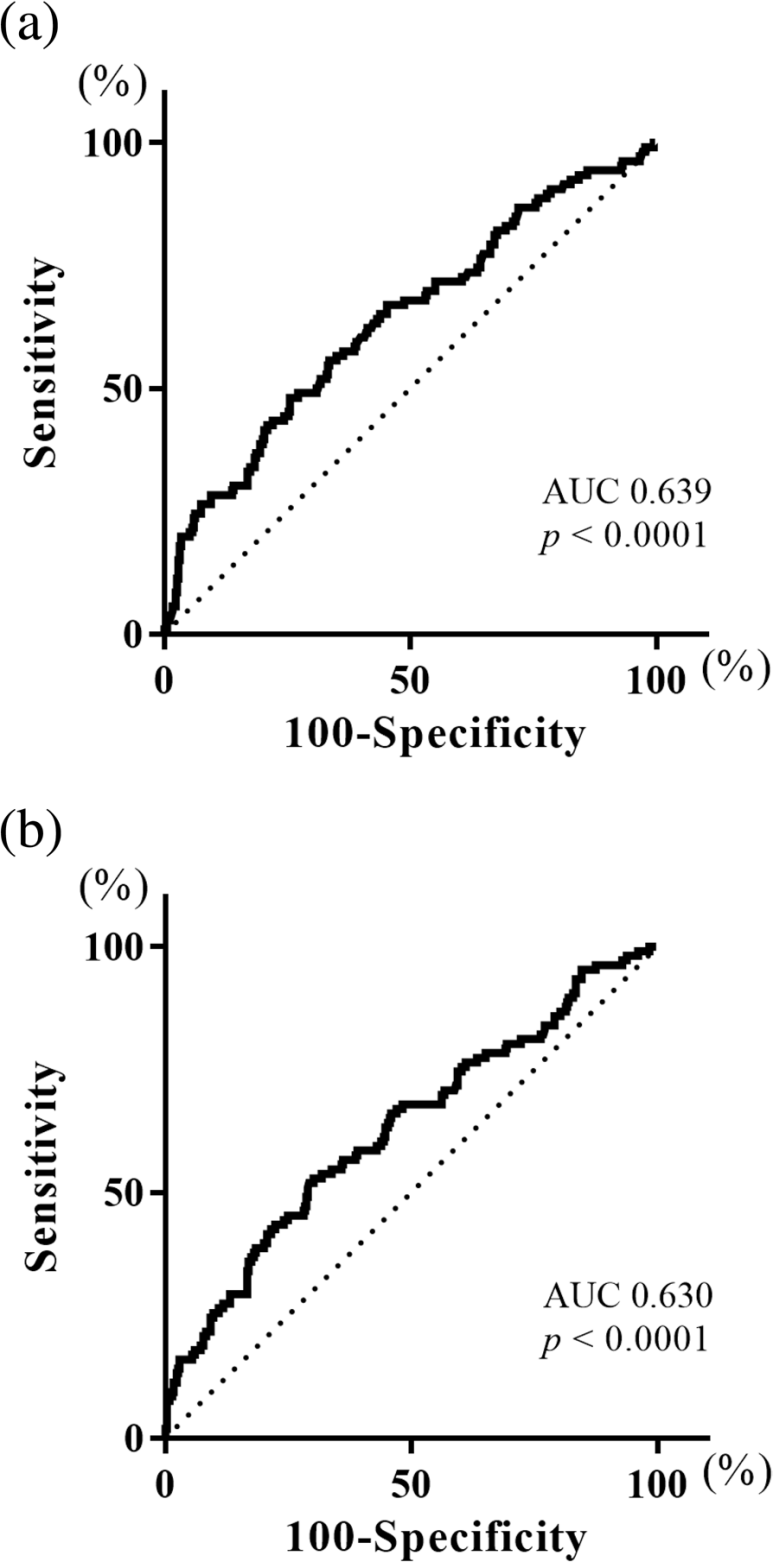
Receiver-operating characteristic (ROC) curve shows the optimal prognostic cutoff for (**a**) preoperative LCR, (**b**) postoperative LCR for the overall survival status

**Fig. 2:**
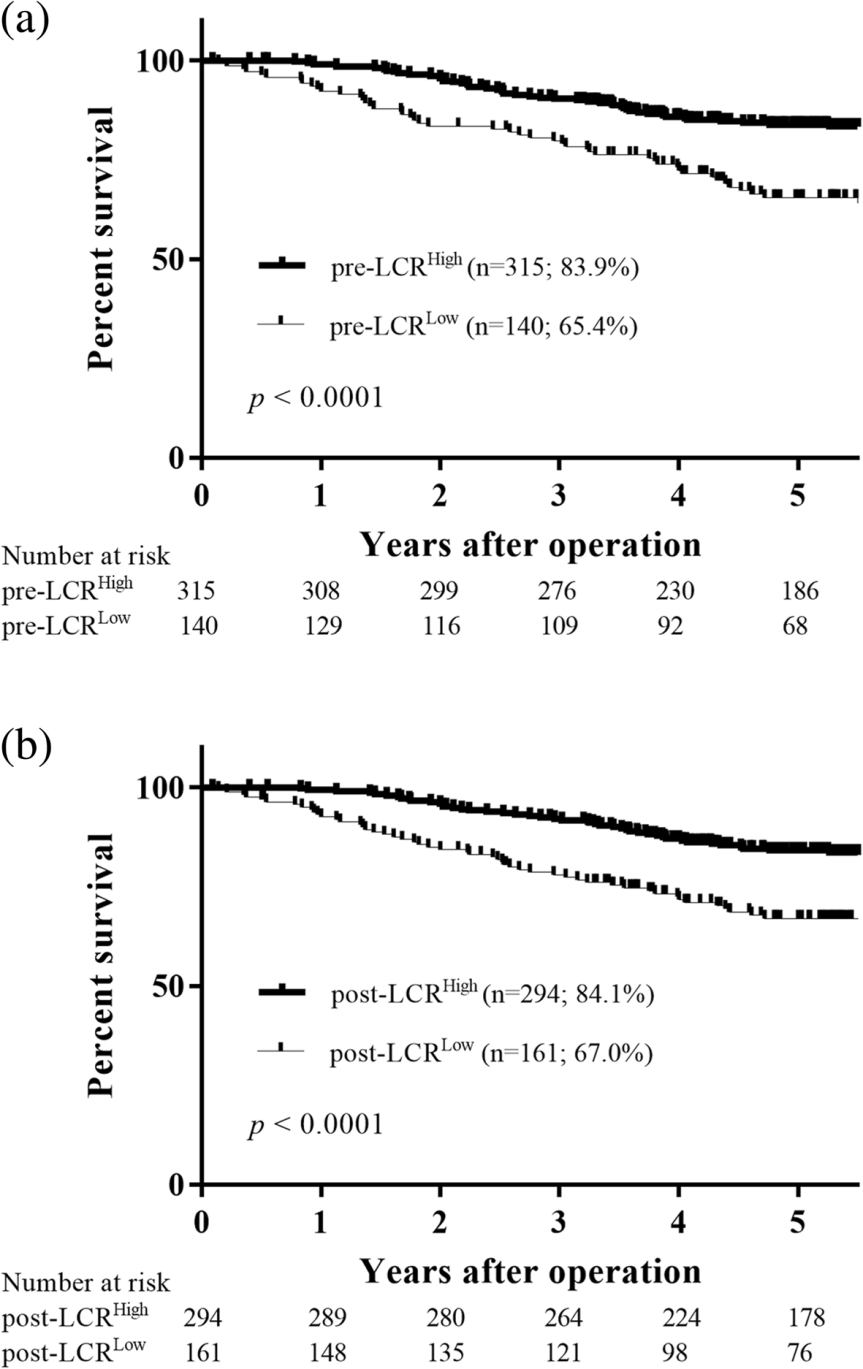
Survival curves according to the preoperative LCR. **a** The 5 year survival rate was significantly worse in patients with pre-LCR^L^°^w^ than in those with pre-LCR^High^ (65.4% vs. 83.9%, *p* < 0.0001). **b** Survival curves according to the postoperative LCR. The 5 year survival rate was significantly worse in patients with post-LCR^L^°^w^ than in those with post-LCR.^High^ (67.0% vs. 84.1%, *p* < 0.0001)

We next investigated the prognostic significance of preoperative and postoperative LCR in patients with GC. Receiver-operating characteristic (ROC) analysis of the OS status showed that the optimal cutoff values of the pre and postoperative LCR were 23,800 (area under the curve [AUC], 0.639; *p* < 0.0001; Fig. 1a) and 13,033 (AUC, 0.630; *p* < 0.0001; Fig. 1b), respectively. Patients were divided accordingly as follows: pre-LCR ≥ 23,800 (pre-LCR^High^, *n* = 315), pre-LCR < 23,800 (pre-LCR^L^°^w^, *n* = 140), post-LCR ≥ 13,033 (post-LCR^High^, *n* = 294), and post-LCR < 13,033 (post-LCR^L^°^w^, *n* = 161). The 5 year OS rates were significantly related to pre-LCR (pre-LCR^High^: 83.9%; pre-LCR^L^°^w^: 65.4%; *p* < 0.0001; Fig. 2a) and post-LCR (post-LCR^High^: 84.1%; post-LCR^L^°^w^: 67.0%; *p* < 0.0001; Fig. 2b).

**Fig. 3:**
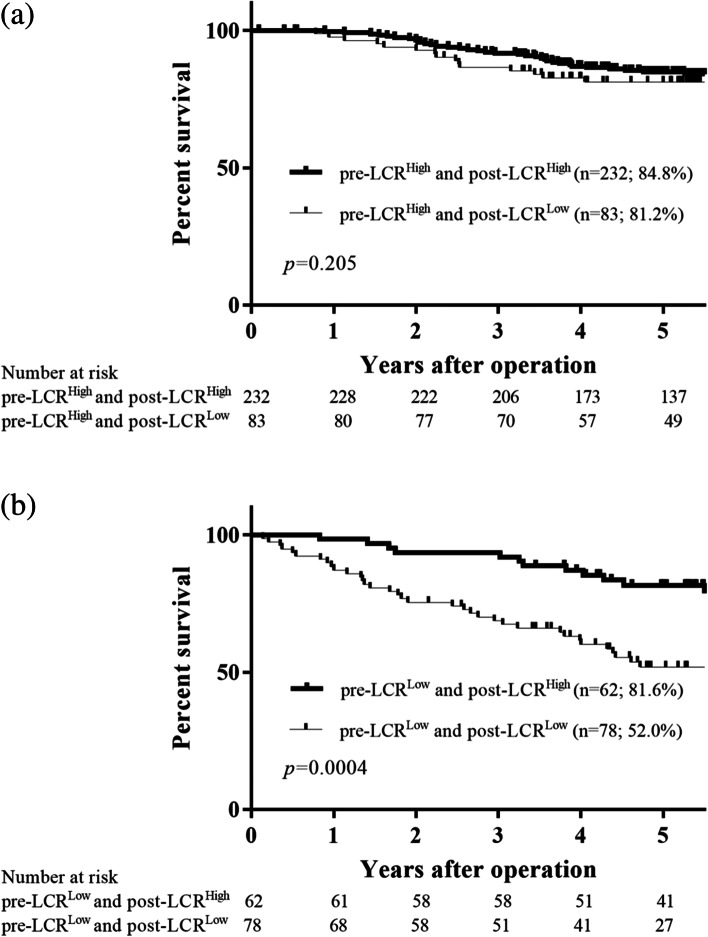
Survival curves according to the postoperative LCR of patients with pre-LCR^High^ (**a**) and those with pre-LCR^L^°^w^ (**b**). The 5 year survival among patients with pre-LCR^High^ and post-LCR^High^ and among those with pre-LCR^High^ and post-LCR^L^°^w^ did not differ significantly (84.8% vs. 81.2%, *p* = 0.205). On the contrary, the 5 year survival rate was significantly worse in patients with pre-LCR^L^°^w^ and post-LCR^L^°^w^ than in those with pre-LCR^L^°^w^ and post-LCR.^High^ (52.0% vs. 81.6%, *p* < 0.0001)

Overall, 232, 83, 62, and 78 patients were in the pre-LCR^High^ and post-LCR^High^, pre-LCR^High^ and post-LCR^L^°^w^, pre-LCR^L^°^w^ and post-LCR^High^, and pre-LCR^L^°^w^, and post-LCR^L^°^w^ groups, respectively. The 5 year OS rates were 84.8% and 81.2% for patients with pre-LCR^High^ and post-LCR^High^ and for patients with pre-LCR^High^ and post-LCR^L^°^w^, respectively, but this difference was not significant (*p* = 0.205; Fig. 3a). On the contrary, the 5 year OS rates were 81.6% and 52.0% for patients with pre-LCR^L^°^w^ and post-LCR^High^ and for patients with pre-LCR^L^°^w^ and post-LCR^L^°^w^, respectively, and this difference was significant (*p* = 0.0004; Fig. 3b).

**Fig. 4:**
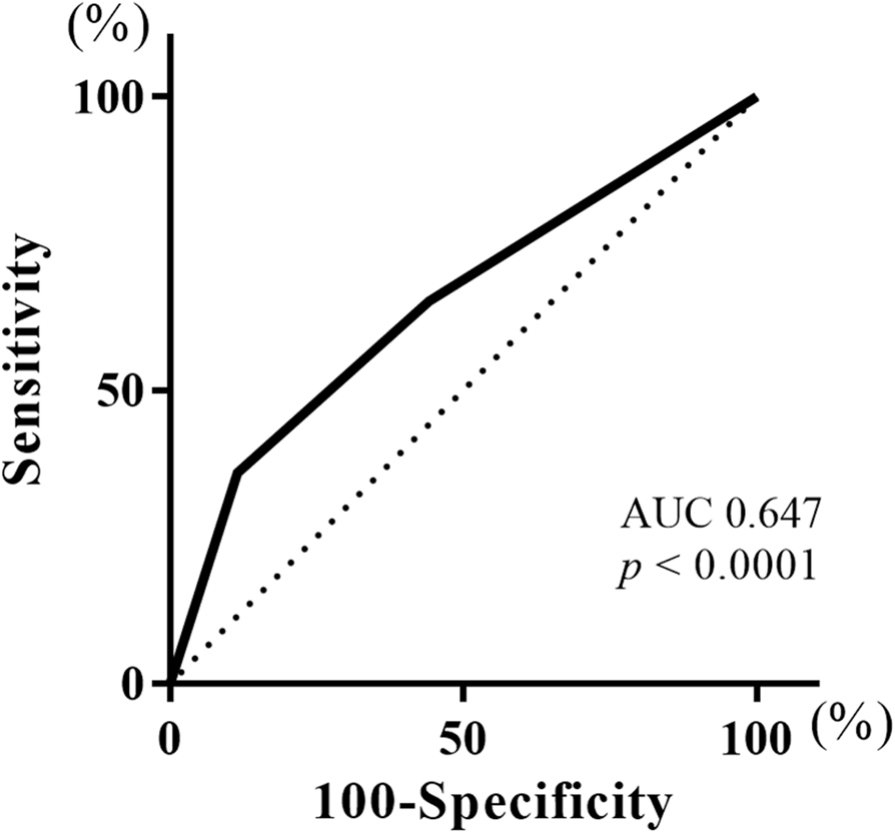
Receiver-operating characteristic (ROC) curve shows the optimal prognostic cutoff for the combination of the preoperative and postoperative LCR for overall survival status

**Fig. 5:**
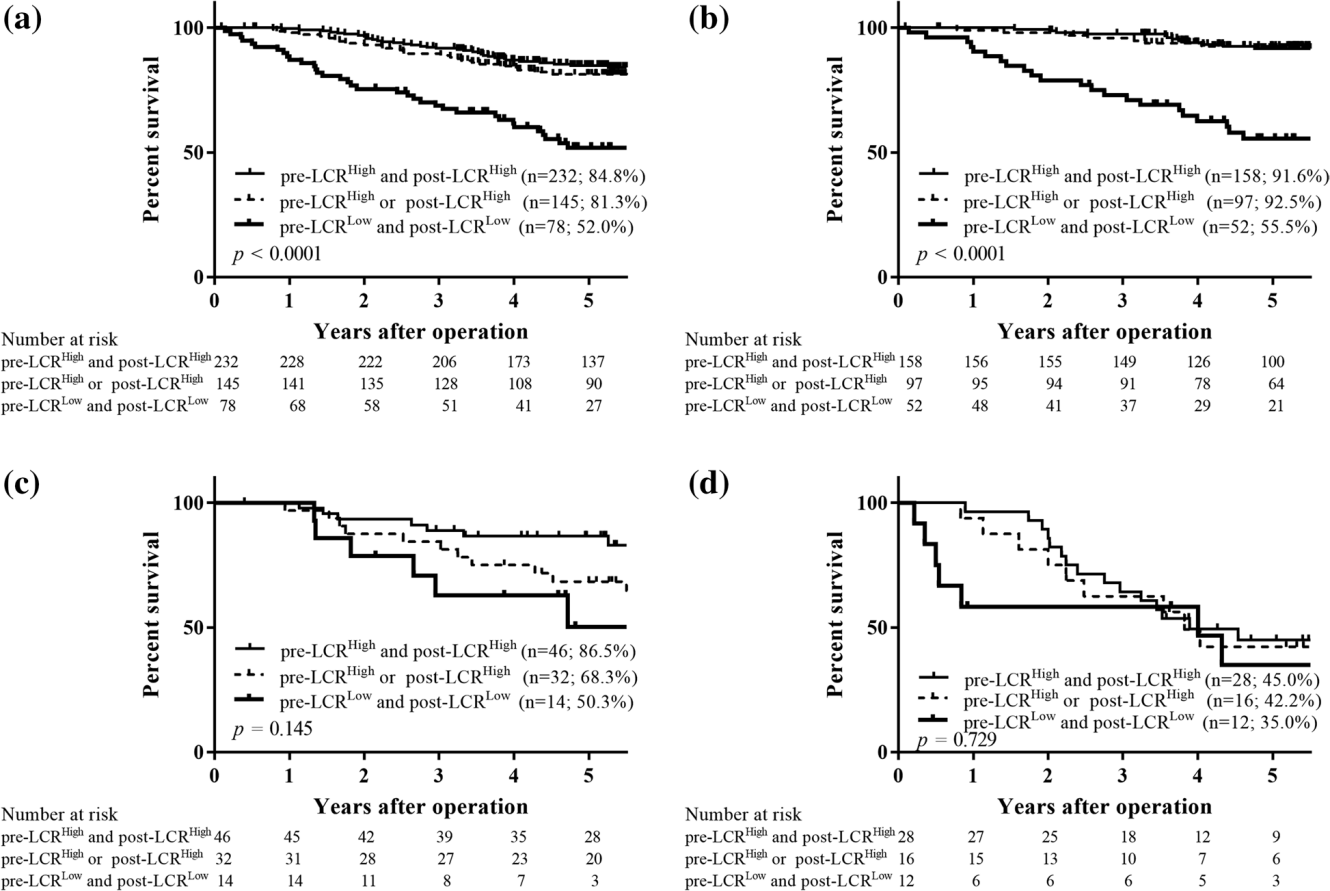
Overall survival curves of all patients (**a**), and those classified into stage I (**b**), stage II (**c**), and stage III (**d**)

**Fig. 6:**
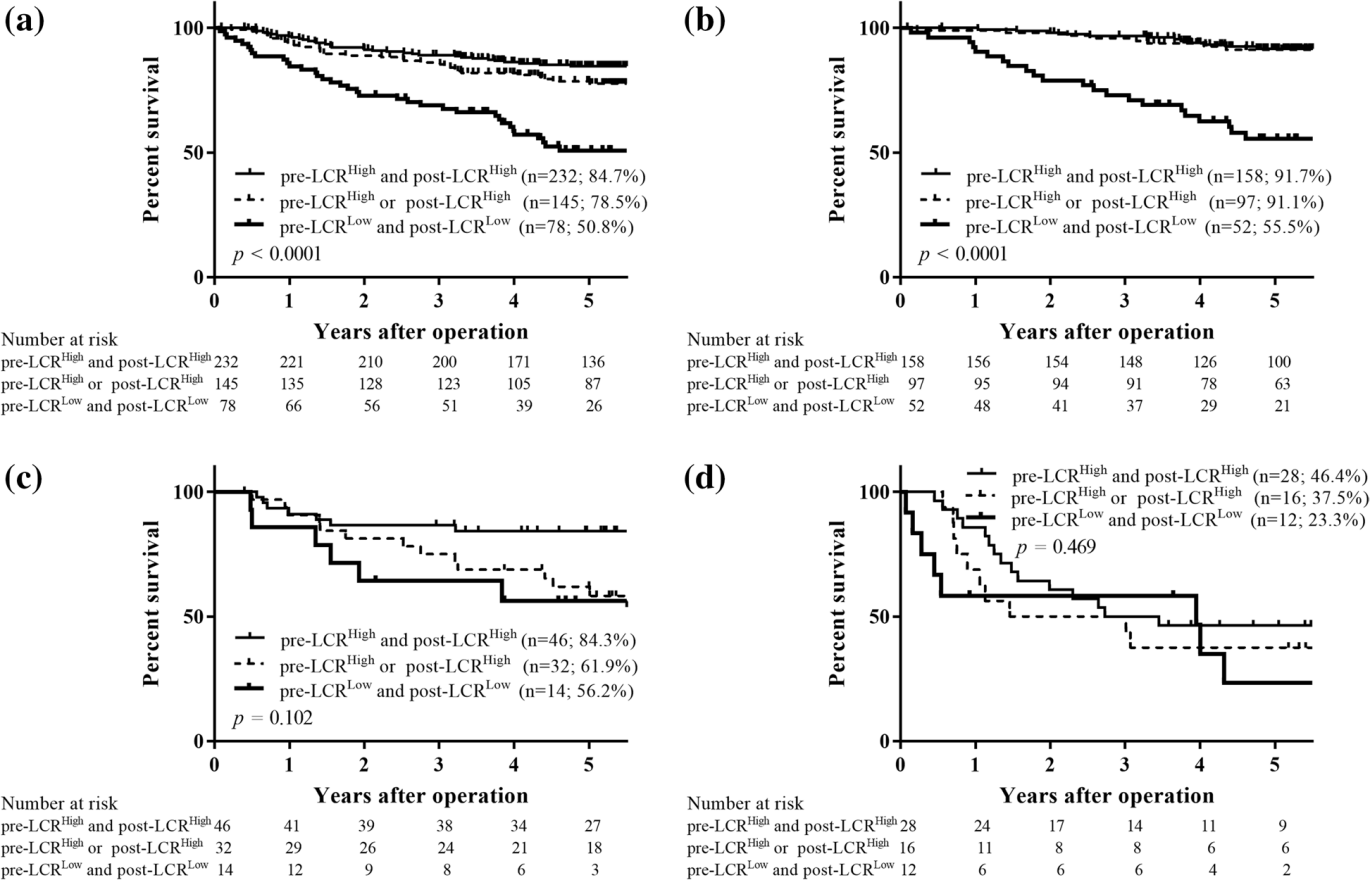
Relapse-free survival curves of all patients (**a**), and those classified into stage I (**b**), stage II (**c**), and stage III (**d**)

The patients were then divided into groups A (those with pre-LCR^High^ and post-LCR^High^), B (those with either pre-LCR^High^ and post-LCR^L^°^w^ or pre-LCR^L^°^w^ and post-LCR^High^), and C (those with pre-LCR^L^°^w^ and post-LCR^L^°^w^). Group B contained two subgroups because the 5 year OS rates were almost the same (81.2% in patients with pre-LCR^High^ and post-LCR^L^°^w^ and 81.6% in those with pre-LCR^L^°^w^ and post-LCR^High^), as mentioned above. The patients in groups A, B, and C were assigned 0, 1, and 2, respectively. ROC curves were constructed for the OS status, and then the AUC values were compared to assess the discrimination ability of the preoperative LCR, postoperative LCR, and the combination of the pre- and postoperative LCR (Fig. 4). Among the three prognostic scores, the combination of the preoperative and postoperative LCR had the highest AUC value (0.647), followed by the preoperative LCR (AUC 0.639) and the postoperative LCR (AUC 0.630). These findings indicate that the combination of preoperative and postoperative LCR was more useful for predicting the prognosis of patients with GC than either the preoperative or postoperative LCR alone. The overall 5 year survival rates were 84.8%, 81.3%, and 52.0% for groups A, B, and C, respectively, and these differences were significant (*p* < 0.0001; Fig. 5a). In a stage-specific analysis, the OS in group C was significantly worse in patients with stage I disease (*p* < 0.0001; Fig. 5b). However, the 5-year OS rates did not significantly differ among patients with stage II and those with stage III disease (stage II, *p* = 0.145, Fig. 5c; stage III, *p* = 0.729, Fig. 5d). Moreover, the relapse-free 5 year survival rates were 84.7%, 78.5%, and 50.8% for groups A, B, and C, respectively, and these differences were significant (*p* < 0.0001; Fig. 6a). Likewise, patients with stage I disease in group C also had a significantly worse RFS (*p* < 0.0001; Fig. 6b). However, the 5-year RFS rates did not differ significantly among patients with stage II and stage III disease (stage II, *p* = 0.102, Fig. 6c; stage III, *p* = 0.469, Fig. 6d).

We conducted a univariate analysis of the clinicopathological factors considered to be prognostic predictors of OS in patients with GC. The univariate analysis identified age, tumor size, depth of invasion, lymph node metastasis, lymphatic and venous involvement, postoperative complications, and the combination of pre- and postoperative LCR as prognostic factors. Then, in the multivariate analysis, we included parameters significant at *p* < 0.05 in the univariate analysis. The multivariate analysis revealed that the combined preoperative and postoperative LCR, age, and lymph node metastasis were independent prognostic indicators of OS (Table 2). Additionally, similar results were observed in the uni- and multivariate analyses of prognostic factors for RFS (Table 3).

**Table 2:** Univariate and multivariate analyses of prognostic factors for overall survival in patients with gastric cancer

Variables	Univariate analysis			Multivariate analysis		
	*p* value	HR ^d^	95% CI ^e^	*p* value	HR	95% CI
Age (≥ 75 vs. < 75)	0.0004	1.996	1.361–2.926	0.002	1.929	1.285–2.896
Gender (Male vs. Female)	0.100	1.489	0.931–2.381			
Tumor size (≥ 4 vs. < 4)	0.0037	1.760	1.202–2.576	0.307	1.247	0.816–1.905
Differentiation (Undifferentiated vs. Differentiated)^a^	0.393	1.181	0.806–1.730			
Depth of invasion (T2–4 vs. T1)^b^	< 0.0001	2.279	1.552–3.347	0.698	1.111	0.653–1.888
Lymph node metastasis (Present vs. Absent)	< 0.0001	2.897	1.978–4.244	0.0001	2.476	1.564–3.919
Lymphatic involvement (Present vs. Absent)	< 0.0001	2.458	1.587–3.807	0.518	0.793	0.393–1.601
Venous involvement (Present vs. Absent)	< 0.0001	2.814	1.844–4.297	0.058	1.908	0.978–3.723
Postoperative complications (Present vs. Absent)^c^	0.002	1.844	1.251–2.717	0.072	1.446	0.967–2.163
Combination of pre and postoperative LCR						
pre- and post-LCR^L^°^w^ vs. pre- or post-LCR^High^	< 0.0001	2.810	1.747–4.519	< 0.0001	2.781	1.719–4.496
pre- and post-LCR^L^°^w^ vs. pre- and post-LCR^High^	< 0.0001	3.831	2.434–6.031	< 0.0001	3.023	1.893–4.829

^a^ Histology: Differentiated, papillary, or tubular adenocarcinoma; undifferentiated, poorly differentiated or mucinous adenocarcinoma, or signet ring cell carcinoma

^b^ Depth of invasion: T1, tumor invasion of the lamina propria or submucosa; T2, tumor invasion of the muscularis propria or subserosa; T3, tumor penetration of the serosa; T4, tumor invasion of adjacent organs

^c^ Postoperative complications were considered grade 2 or higher according to the Clavien–Dindo classification

^d^ *HR* Hazard ratio

^e^ *CI* Confidence interval

**Table 3:** Univariate and multivariate analyses of prognostic factors for relapse-free survival in patients with gastric cancer

Variables	Univariate analysis			Multivariate analysis		
	*p* value	HR^d^	95% CI^e^	*p* value	HR	95% CI
Age (≥ 75 vs. < 75)	0.0026	1.777	1.222–2.585	0.011	1.669	1.124–2.477
Gender (Male vs. Female)	0.092	1.483	0.937–2.347			
Tumor size (≥ 4 vs. < 4)	0.003	1.759	1.212–2.553	0.495	1.156	0.763–1.750
Differentiation (Undifferentiated vs. Differentiated)^a^	0.321	1.208	0.832–1.754			
Depth of invasion (T2–4 vs. T1)^b^	< 0.0001	2.536	1.741–3.694	0.453	1.220	0.726–2.051
Lymph node metastasis (Present vs. Absent)	< 0.0001	3.388	2.333–4.920	< 0.0001	2.757	1.756–4.329
Lymphatic involvement (Present vs. Absent)	< 0.0001	2.602	1.695–3.994	0.422	0.756	0.382–1.497
Venous involvement (Present vs. Absent)	< 0.0001	3.006	1.985–4.553	0.041	1.960	1.028–3.735
Postoperative complication (Present vs. Absent)^c^	0.002	1.816	1.244–2.652	0.047	1.487	1.006–2.199
Combination of pre and postoperative LCR						
pre- and post-LCR^L^°^w^ vs. pre- or post-LCR^High^	0.0001	2.474	1.566–3.906	0.0002	2.398	1.511–3.805
pre- and post-LCR^L^°^w^ vs. pre- and post-LCR^High^	< 0.0001	3.845	2.450–6.031	< 0.0001	3.120	1.961–4.965

^a^ Histology: Differentiated, papillary, or tubular adenocarcinoma; undifferentiated, poorly differentiated or mucinous adenocarcinoma, or signet ring cell carcinoma

^b^ Depth of invasion: T1, tumor invasion of the lamina propria or submucosa; T2, tumor invasion of the muscularis propria or subserosa; T3, tumor penetration of the serosa; T4, tumor invasion of adjacent organs

^c^ Postoperative complications were considered grade 2 or higher according to the Clavien–Dindo classification

^d^ *HR* Hazard ratio

^e^ *CI* Confidence interval

**Fig. 7:**
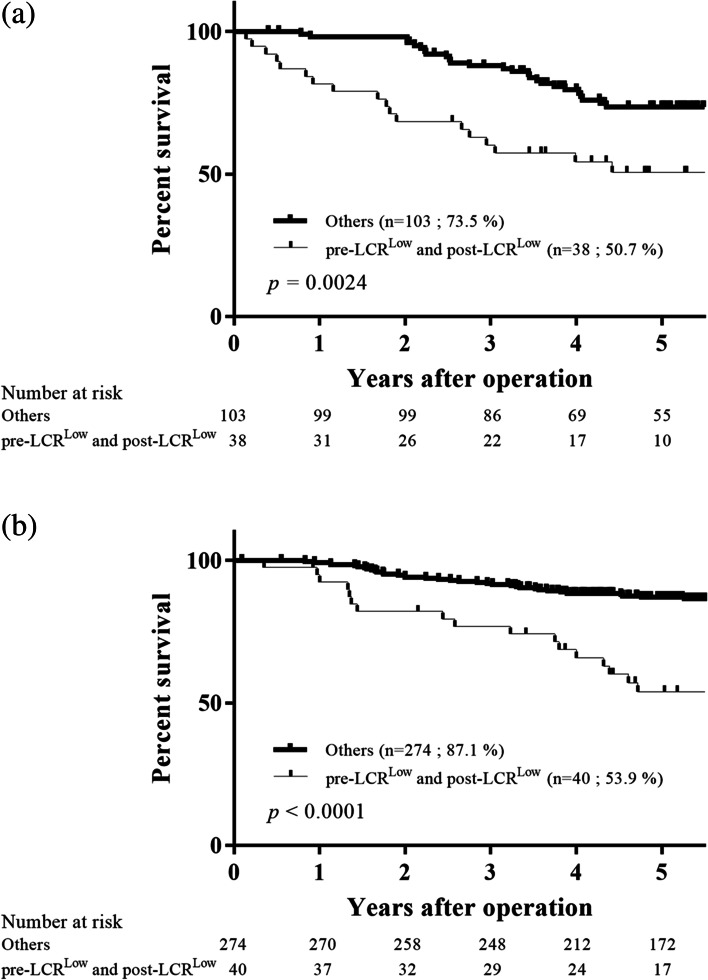
Survival curves according to the pre- and postoperative LCR values of patients with complications (**a**) and those without complications (**b**). The overall 5-year survival rate was significantly worse in patients with pre-LCR^L^°^w^ and post-LCR^L^°^w^ regardless of the presence or absence of complications (*p* = 0.0024, *p* < 0.0001, respectively)

**Fig. 8:**
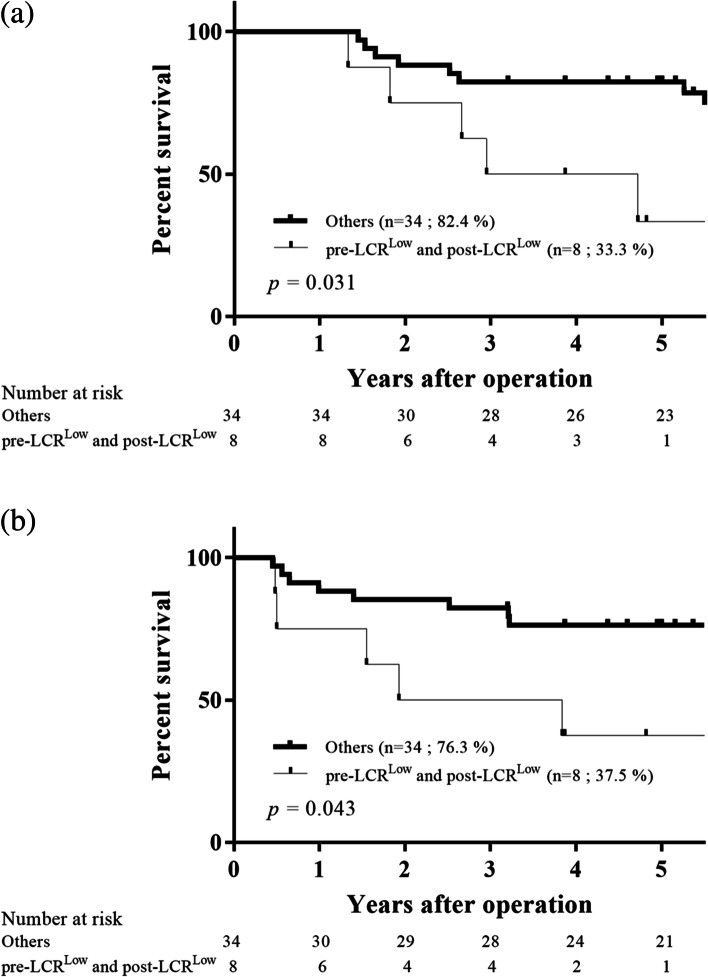
Survival curves according to the pre- and postoperative LCR values of patients with stage II/III gastric cancer who were ineligible for adjuvant chemotherapy, particularly those with pT1N2-3b and pT3N0. The overall (**a**) and relapse-free (**b**) 5-year survival rates were significantly worse in those with pre-LCR^L^°^w^ and post-LCR^L^°^w^ than in other patients (*p* = 0.031 and *p* = 0.043, respectively)

According to the abovementioned results, we constructed additional considerations focused on the clinical impact of the pre- and postoperative low LCR values (pre-LCR^Low^ and post-LCR^Low^) in patients with GC. First, given that LC and CRP levels, which are LCR components, are both very sensitive indicators that are influenced by a patient's physical status, such as inflammation caused by complications, we analyzed the prognosis of patients with and without complications to eliminate such influence. The prognosis was significantly worse in patients with pre-LCR^Low^ and post-LCR^Low^ regardless of the presence or absence of complications (*p* = 0.0024, presence of complication, Fig. 7a; *p* < 0.0001, absence of complication, Fig. 7b). Second, according to the Japanese guideline, patients with stage II/III GC, except for those with pT1N2-3b/pT3N0, are targets for adjuvant chemotherapy as a standard postoperative treatment [17]. However, some patients with pT1N2-3b/pT3N0 GC assigned to the surgery-alone group had a poor prognosis. Hence, we examined whether using pre- and postoperative LCR values can clearly identify the poor-prognosis group in patients with pT1N2-3b/pT3N0 GC who may be appropriate candidates for adjuvant chemotherapy. Among the 42 patients with pT1N2-3b/pT3N0 GC who were ineligible for adjuvant chemotherapy in the present study, the prognosis was significantly worse in those with pre-LCR^Low^ and post-LCR^Low^ than in other patients (*p* = 0.031, OS, Fig. 8a; *p* = 0.043, RFS; Fig. 8b).

## Discussion

There have been substantial discussions about the importance of the systemic inflammation that results from the complex interactions between cancer and host during disease development [8, 18]. Furthermore, accumulating evidence has demonstrated the association between poor nutritional status and impaired immunity in patients with cancer [19, 20]. In relation to these findings, LCR has been developed as a nutrition-inflammation marker and serves as a prognostic biomarker in patients with GC [12]. However, it is still unclear how the LCR is associated with the prognosis of patients with cancer. With respect to the association between LC and the prognosis of patients with cancer, lymphocytes, which include CD4^+^ and CD8^+^ T cells, natural killer (NK) cells, NKT cells, gamma-delta T cells, and B cells, have been reported to play an important role in tumor immunity in the host. In this connection, functional impairment of lymphocytes and decreased lymphocyte counts have been reported in various types of cancers [21, 22], and this is likely associated with impairment of antitumor immunity. Furthermore, Saito et al. reported that low preoperative and postoperative LC values were significantly associated with poor prognosis of patients with GC [23]. Regarding the association between CRP and the prognosis of patients with cancer, CRP is the most common marker used to evaluate the magnitude of systemic inflammation because of its sensitivity, specificity, and reproducibility of analysis in hospital laboratories. Additionally, CRP is an acute-phase reactant synthesized predominantly in the liver [24] that is regulated by proinflammatory cytokines, particularly IL-6 [25]. Kim et al. reported that in GC, serum IL-6 levels were positively correlated with CRP levels and were also correlated with the TNM stage; the CRP level served as a poor prognostic factor for disease recurrence and OS [26]. Considering these findings, the LCR is likely to be associated with tumor progression and the prognosis of patients with cancer.

In the present study, we demonstrated that preoperative LCR was significantly associated with tumor-related factors, such as tumor size, depth of invasion, and lymph node metastasis, among others, and that a low preoperative LCR was related to an unfavorable prognosis. These findings are consistent with those of a previous report [12] and could be considered consequences of complex host–tumor interactions that play a pivotal role in GC development. On the contrary, a low postoperative LCR was significantly associated with poor prognosis despite the lack of association between postoperative LCR and tumor-related factors. Interestingly, similar results have also been reported in previous studies, which found that other postoperative markers, such as the postoperative NLR and PNI, served as poor prognostic factors despite the lack of correlation between these markers and tumor-related factors [10, 11, 27, 28]. Naturally, the postoperative markers were not associated with tumor-related factors considering the tumors had been removed. However, the detailed mechanism by which postoperative markers, including the postoperative LCR, are associated with the prognosis of patients with cancer remains unclear. Presumably, one possible mechanism is the effect of micro metastatic residual tumor cells that cannot be eradicated by surgery. Complete surgical resection of the primary tumor is performed to achieve a cure for locally advanced GC, but even after complete resection, tumor recurrence can still occur [29]. The cause of postoperative tumor recurrence is considered an effect of micro metastasis, which exists outside the surgical field and gradually multiplies to affect host survival. Some studies have claimed that the immunological response against infectious postoperative complications enhances the viability of micro metastatic residual tumor cells after surgery [30–32]. Additionally, noninfectious postoperative complications, such as anastomotic stenosis, lymphorrhea, and bleeding, induce malnutrition and cause lymphopenia, which results in immunosuppression [10]. Through the above mechanisms, micro metastatic residual tumor cells secrete various proinflammatory cytokines and negative immune modulators; these triggers decreased postoperative LC and increased postoperative CRP, which might be responsible for a low postoperative LCR. Therefore, a low postoperative LCR is associated with a poor prognosis. Actually, according to the present study, low preoperative and postoperative LCR was significantly associated with the occurrence of postoperative complications and was an independent prognostic indicator of OS and RFS.

With respect to the prognostic utility of preoperative LCR in patients with GC, as reported previously, our results indicated that low preoperative LCR was significantly associated with a poor prognosis. Similarly, low postoperative LCR was also significantly associated with a poor prognosis, which was a novel finding. Considering these results, it can be presumed that perioperative low LCR, namely, a continuous systemic inflammatory response and suppression of the entire immune system after surgery of a patient with cancer, creates a favorable environment for micro metastatic growth. Thus, we hypothesized that the combination of the preoperative and postoperative LCR might be more useful in the prediction of the prognosis of patients with GC than either the preoperative LCR or the postoperative LCR alone. Actually, considering the result of the comparison of the AUC value for the OS status, the combination of the preoperative and postoperative LCR more precisely predicted a poor prognosis than either the preoperative or postoperative LCR alone. Additionally, the multivariate analysis revealed that the combination of the low preoperative and postoperative LCR was an independent prognostic indicator of OS. Furthermore, Fig. 8 shows that the prognosis of patients with pT1N2-3b/pT3N0 GC with low pre- and postoperative LCR values treated by surgery alone may be poor and could be candidates for adjuvant chemotherapy. With regard to using pre- and postoperative LCR values for perioperative management, a high preoperative LCR correlates with a good prognosis regardless of a high or a low postoperative LCR; thus, an aggressive preoperative nutritional therapy to improve the nutritional status of patients with a low LCR can increase the LCR value, thereby prolonging the prognosis (Fig. 3). Moreover, patients with high postoperative LCR values but low preoperative LCR values may still have a good prognosis, suggesting that performing surgery without complications and providing nutritional therapy early in the postoperative period may prolong the prognosis. Therefore, although the AUC of the ROC curve in this study exploring the prognostic utility of the combination of the pre- and postoperative LCR values were relatively low, we found certain new findings that can help clinicians who are involved in GC treatment make appropriate treatment decisions. Taken together, both the post- and preoperative statuses are important when considering patients’ prognosis.

Over the past few years, many researchers have suggested that the number of circulating tumor cells (CTCs) could be a prognostic indicator in patients with cancer, such as breast cancer, small-cell lung cancer, colorectal cancer, and GC [33–36]. However, the techniques by which CTCs are detected are often complicated and unsuitable for routine clinical settings. Conversely, inflammation and nutrition markers including perioperative LCR are easy to measure and are useful for prognostic prediction. Moreover, Zheng et al. demonstrated that preoperative markers, such as the systemic immune-inflammation index (SII), NLR, PLR, and PNI, are robust predictors of CTCs in patients with GC undergoing tumor resection [37]. A further study on whether postoperative markers including postoperative LCR are associated with CTC detection in patients with GC who have undergone tumor resection or not should be conducted.

This study also has some limitations. First, some bias was present because of the study’s retrospective nature. Second, we measured the LCR 1 month after surgery and considered that value to be the postoperative LCR; however, the appropriate timing of when the postoperative LCR should be measured remains unclear. Third, all the patients enrolled in this study were from a single institution in Japan. Fourth, the pre- and postoperative LCR values were only assessed in a relatively small number of patients because of the lack of laboratory data. To overcome these limitations, a large-scale, prospective randomized controlled trial is needed.

## Conclusion

The combination of preoperative and postoperative LCR appears to be useful to predict the prognosis of patients with GC. Since perioperative assessment of LC and CRP is readily available, noninvasive, and easy to perform, measurement of the pre- and postoperative LCR may be useful as a clinical biological tool in routine clinical settings.

## Data Availability

The datasets used and analyzed during the current study are not publicly available because some contents could compromise the anonymity of research participants. However, they are available from the corresponding author on a reasonable request.

## Abbreviations

LCR: Lymphocyte-C-reactive protein ratio
GC: Gastric cancer
NLR: Neutrophil–lymphocyte ratio
PLR: Platelet-lymphocyte ratio
GPS: Glasgow Prognostic Score
PNI: Prognostic nutritional index
LC: Lymphocyte count
CRP: C-reactive protein
OS: Overall survival
RFS: Relapse-free survival
NK: Natural killer
CTC: Circulating tumor cell
SII: Systemic immune-inflammation index
